# Indication for pharmacological treatment is often lacking: a cross-sectional study on the quality of drug therapy among the elderly

**DOI:** 10.1186/s12877-015-0117-x

**Published:** 2015-10-08

**Authors:** Jessica Skoog, Patrik Midlöv, Anders Beckman, Jan Sundquist, Anders Halling

**Affiliations:** Department of Clinical Sciences in Malmö, Center for Primary Health Care Research, Lund University, SE-205 02 Malmö, Sweden; Stanford Prevention Research Center, Stanford University School of Medicine, Stanford, CA USA; Research Unit of General Practice, Department of Public Health, University of Southern Denmark, J.B. Winsløws Vej 9A, DK-5000 Odense C, Denmark

**Keywords:** Pharmacological treatment, Drug therapy, Elderly patients, Aged patients, Indication, Prescription drugs, Potential inappropriate medicine, Discontinue treatment

## Abstract

**Background:**

Although the elderly have a substantially higher drug use than younger patients, even after adjustment for multimorbidity, there is limited knowledge about the elderly’s indication for treatment. It is essential for elderly patients to have a well-planned drug therapy. The first step towards a correct and safe drug therapy is to ensure that the patient’s drugs have an indication, i.e. correct diagnoses are linked to all of the prescription drugs. The aim of this study was to examine to what extent elderly patients have indication for a number of their prescribed drugs and, furthermore, if there are any differences in indication for treatment depending on gender, age, level of multimorbidity and income.

**Method:**

Data were collected on individuals aged 65 years or older in Östergötland County in Sweden. To estimate the individual level of multimorbidity the Johns Hopkins ACG Case-Mix System was used. A report from the Swedish National Board of Health and Welfare was used to identify prescription drugs, for which it is important to have a correct diagnosis. The proportions of patients having indication for these prescription drugs were calculated. Odds ratios of having indication for treatment depending on gender, age, multimorbidity level and income were calculated.

**Results:**

On average 45.1 % (range 12.9 % – 75.8 %) of the patients’ prescribed drugs had indication. Proton pump inhibitors were associated with the lowest level of indication (12.9 %) and digoxin was associated with the highest level of indication for treatment (75.8 %). Patients aged 80 years or older had the lowest odds ratios of having indication for treatment.

**Conclusion:**

On average, there was indication for treatment in less than half of the prescription drugs studied. The quality was highest in relation to multimorbidity and lowest in relation to age. The result may to some extent be explained by substandard registration of diagnoses. Since lack of quality of prescription drug use is highly associated with inconvenience among the elderly, as well as high costs to society, it is important that future research and allocation of resources focus on the quality of elderly patients’ drug therapy.

## Background

The population in the Western countries is ageing [1]. Chronic illness is more common at older age [2] and is often associated with increased pharmacological treatment among the elderly [3]. After adjustment for multimorbidity level the use of prescription drugs has been shown to still be substantially higher among older patients compared to younger [4]. There are at least two reasons for a remaining age difference after adjustment for multimorbidity level: 1. Medical reasons like progression of diseases not reflected by the labelling of the diagnosis, for example diabetes and heart failure [5, 6], 2. ‘The prescribing cascade’ which is described as side effects of a prescription drug, which are misinterpreted as a new medical condition leading to prescription of a new drug [7]. An additional explanation could be that the use of prescription drugs is not properly planned, evaluated and discontinued among the elderly patients, which may lead to an unnecessarily high use of prescription drugs.

Treatment of elderly patients with prescription drugs puts high demands on the prescribers. Changes in pharmacokinetics and pharmacodynamics make elderly patients more sensitive to side effects [8]. The elderly patients often use many prescription drugs, and this increases the risk of adverse drug reactions [9]. Because of the above it is essential that elderly patients have a well-thought-out pharmacological treatment. Already in the 1980s, WHO started their work on improving the pharmacological treatment among elderly patients, and in 1997 the first report was published on this topic [10]. One of the first steps towards the correct and safe use of prescription drugs among elderly patients is to ensure that the prescription drugs used by the patients have an indication, i.e. correct diagnoses are linked to all of the prescription drugs [10]. In order to improve the quality of prescription drug use among elderly patients, efforts have been made to define criteria for appropriate prescription drug use. The most widespread criteria are American Geriatrics Society Beers Criteria for Potentially Inappropriate Medication Use in Older Adults (Beers Criteria) [11]. The Beers Criteria serve as a guide to physicians and list potential inappropriate medications that ought to be avoided among elderly patients to reduce polypharmacy and adverse drug reactions. However, many of the listed prescription drugs in the Beers Criteria are not available in Europe, and prescribing behaviour and clinical guidelines differ from the USA. Therefore, many European countries have developed criteria of their own [12–14]. In Sweden, the National Board of Health and Welfare authored a report about satisfactory pharmacological treatment among elderly patients [15]. One of the aims of the report was to serve as support to physicians when prescribing drugs to elderly patients. Among other things the report resulted in a list of prescription drugs, for which it is particularly important to have accurate and up-to-date diagnoses, see Table 1.

**Table 1:** Prescription drugs for which an accurate and up-to-date diagnosis is particularly important in elderly patients^a^

Prescription drugs	
Antipsychotic drugs	
Proton pump inhibitors	
Digoxin	
Loop diuretics	
Serotonin reuptake inhibitors	
Cox-inhibitors (NSAIDs)	
Paracetamol	
Opioids	

^a^According to the Swedish National Board of Health and Welfare. Available at: http://www.socialstyrelsen.se/lists/artikelkatalog/attachments/18085/2010-6-29.pdf

The main objective of this study was to examine to what extent elderly patients have diagnoses linked to a number of their prescription drugs, i.e. indication for treatment, identified by the Swedish National Board of Health and Welfare as potential inappropriate medication. Since former research has shown that females, elderly, multimorbid patients and patients with low socioeconomic status use more prescription drugs and have higher levels of polypharmacy [3, 16, 17], we also wanted to examine whether there are any differences in having indication for these prescription drugs according to gender, age, level of multimorbidity and income.

## Methods

### Study population

Data were collected from the total population aged 65 years or older in Östergötland County in Sweden, having about 400 000 residents in 2006. Östergötland is situated 200 km southwest of Stockholm, and the age demographics match that of the rest of Sweden [18]. Data were obtained from the Care Data Warehouse in Östergötland (CDWÖ), a register containing information on both public and private care. The CDWÖ register was established in 1998 and contain data on the patients’ diagnoses according to ICD-10, personal identification number and healthcare unit visited from each consultation, i.e. visits at PHCs, hospitals and inpatient care, in Östergötland County. Data are transferred from Östergötland County to the CDWÖ register once every month. Data from the CDWÖ register were collected during 2005–2006. This register has been described previously [19]. The study was approved by the research ethics committee at Linköping University (approval numbers 147/05 and 29/06).

### Independent variables

Multimorbidity was estimated using the Johns Hopkins Adjusted Clinical Groups (ACG) Case-Mix System, a system based on the theory that clustering of morbidity is a better predictor of healthcare costs than the presence of specific diagnoses. By using different building blocks every individual is, at the end, assigned a certain ACG, which corresponds to a certain need for healthcare resources. The system is based on the patients’ diagnoses recorded during a defined period of time. The etiology, duration, severity, method of diagnosis, treatment and need of specialised care are considered for each of the patients’ diagnoses. The ACGs may furthermore be clustered into Resource Utilization Bands (RUBs). Individuals without need of health care according to the ACG Case-Mix system are placed in RUB 0, and individuals with a very high degree of need for healthcare resources are placed in RUB 5. For example, preventive interventions correspond to RUB 1, a single chronic diagnosis could correspond to RUB 3 and a certain combination of chronic diagnoses corresponds to RUB 4 or RUB 5. In RUB levels 0–2 there ought to be no patients with pharmacological treatment, and therefore the results from RUB levels 0–2 are not reported in this study. The ACG Case-Mix System has previously been described [20–23]. Information on diagnoses were obtained from the CDWÖ register during 2006.

We chose to define elderly patients as being 65 years or older. The elderly patients were further divided into 65–79 years and 80 years and above.

We used income as a measure of socioeconomic status, reflecting the economic conditions the patients lived in at the time of the study. Education may also be used as a socioeconomic variable, but data on this were incomplete among the elderly patients. The individual disposable income in the study population was divided into quartiles, from the lowest to the highest with equal number of individuals in each quartile. The individual income includes earnings from employment and business, and transfer income (e.g. pension payments etc.), but not capital returns. Data on income were obtained from Statistics Sweden.

### Outcome variable

The proportion of patients with a correct diagnosis linked to the prescription drug, i.e. the proportion of patients having indication for treatment, was the outcome. Information about the use of prescription drugs at the individual level was acquired from the Swedish Prescribed Drug Register, which is maintained by the National Board of Health and Welfare [24]. This register collects information from the National Corporation of Swedish Pharmacies (Apoteket AB) on prescription drugs dispensed from the pharmacies. If a drug was prescribed by a physician, but not collected by the patient, the drug will not be included in the Swedish Prescribed Drug Register. In 2006 the National Corporation of Swedish Pharmacies had a monopoly on sales of prescription drugs, and all prescription drugs were tracked through the National Corporation of Swedish Pharmacies.

The Anatomical Therapeutic Chemical (ATC) classification system was elaborated by WHO to enable internationally comparable studies on prescription drugs [25]. Active substances are classified in different groups according to the organ or system on which they act, and their therapeutic, pharmacological and chemical properties. The drugs are divided into 14 main ATC groups, and these groups are subsequently divided into five levels.

Over-the-counter drugs were not included in this study.

### Statistics

We used a report from the Swedish National Board of Health and Welfare regarding pharmacological treatment among elderly patients [15]. The report establishes that having indications for the prescription drugs is fundamental for satisfactory pharmacological treatment, implying that there is a purpose with the prescription. A number of prescription drugs, for which it is particularly important to have an accurate and up-to-date diagnosis, are listed. These prescription drugs were selected by the Swedish National Board of Health and Welfare since there is a history of prescribing these drugs without a correct indication and, furthermore, because adverse drug reactions are highly associated with these drugs among the elderly. We have used this list from the report in our study. For every one of the selected prescription drugs the diagnoses that were validated as accurate were classified by using the Swedish database of prescription drugs, containing information about indications for all approved prescription drugs in Sweden, see Table 2 [26].

**Table 2:** Prescription drugs for which an accurate and up-to-date diagnosis is particularly important and their valid accurate diagnoses, classified by using the Swedish database of prescription drugs [26]

Prescription drugs	ATC^a^ codes	Accurate diagnoses	ICD-10^b^ codes
Antipsychotic drugs	N05A excluding N05AN	Schizophrenia	F22
			F23
			F24
			F25
		Bipolar disorder	F31
		Severe aggression in Alzheimer’s disease	F91
			G30/F00
Proton pump inhibitors (PPIs)	A02BC	Gastroesophageal reflux	K21
		Stomach ulcer	K25
		Ulcer in the duodenum	K26
		Ulcer in stomach or duodenum	K27
Digoxin	C01AA05	Heart failure	I50
		Atrial fibrillation	I48
Loop diuretics	C03C	Pulmonary oedema	J81
		Heart failure	I50
		Hypertension	I10
		Oedema	R60
		Kidney failure	N18
		Liver failure	K72
Serotonin reuptake inhibitors (SSRIs)	N06AB, N06AX	Depression	F31
			F32
			F33
		Social anxiety disorder	F40
		Panic disorder	F41
		Obsessive-compulsive disorder	F42

^a^Anatomical Therapeutical Classification elaborated by the World Health Organisation. Available at http://www.whocc.no/atcddd

^b^International Statistical Classification of Diseases and Related Health Problems, 10th Revision

The proportion of patients with a correct diagnosis linked to a prescription drug was analysed. The information on diagnoses were obtained from the CDWÖ register during 2005–2006. The data were furthermore analysed in different strata: for males and females alone, for different age levels, for each multimorbidity level and for each income level. The outcome was compared using chi^2^-test. Multimorbidity level 3 was compared with multimorbidity levels 4 and 5 one by one, and income level 1 was compared with income levels 2, 3 and 4 one by one.

Logistic regression was used to examine the odds ratio of having indication for the prescription drugs, giving odds ratios (ORs) and 95 % confidence intervals (CIs). We generated a model that was adjusted for gender, age, multimorbidity level and income level.

The list from the Swedish National Board of Health and Welfare originally also contained cox-inhibitors (NSAIDs), paracetamol and opioids, but since it is very hard to tell which diagnoses that are validated as accurate, not leaving any out, for example cancer or other chronic painful diseases, we excluded these prescription drugs from the analyses.

## Results

The proportion of patients with a correct diagnosis linked to the prescription drug was examined in the study population, comprised by 77 978 individuals aged 65 years or older. Further characteristics are described in Table 3. A total of 44 600 patients collected at least one of the five examined prescription drugs.

**Table 3:** Characteristics of the study population

Variables	N (%)
Gender	
Males	33994 (44 %)
Females	43983 (56 %)
Age	
65-79	51945 (67 %)
80+	26033 (33 %)
Multimorbidity level	
3	35190 (45 %)
4	7107 (9 %)
5	2447 (3 %)
Income level	
1	19495 (25 %)
2	19494 (25 %)
3	19494 (25 %)
4	19494 (25 %)

On average 45.1 % (range 12.9 % – 75.8 %) of the patients’ dispensed drugs examined in this study had correct diagnoses, i.e. indications. Patients 80 years and above had indication for the prescription drugs to the lowest extent (40.8 %), and patients in RUB level 5 had indication to the highest extent (52.2 %).

### Antipsychotic drugs

Of the patients who used antipsychotic drugs 18.0 % (467 of 2 601) had indication for these prescription drugs. There was no difference between the genders or between different multimorbidity levels (RUBs). There was an age difference, where the oldest patients to a lesser extent had indication for the dispensed antipsychotics. There was also an income difference where the poorest patients to a lesser extent had indication for treatment (Table 4).

**Table 4:** Proportion of patients with correct diagnoses linked to the prescribed drugs

	Antipsychotics	Proton pump inhibitors	Digoxin	Loop diuretics	Serotonin reuptake inhibitors
Gender					
Male	17.4 % (156/898)	12.9 % (664/5149)	78.2 % (1080/1381)	70.9 % (4581/6460)	39.2 % (1035/2641)
Female	18.3 % (311/1703)	12.9 % (1010/7853)	73.9 % (1315/1780)	67.9 % (6878/10137)	40.8 % (2570/6298)
Age					
65-79	21.7 % (248/1143)	13.6 % (1077/7894)	78.2 % (921/1178)	69.5 % (4971/7157)	45.8 % (2130/4655)
80+	15.0 %* (219/1458)	11.7 %* (597/5108)	74.3 % (1474/1983)	68.7 % (6488/9440)	34.4 %* (1475/4284)
Multimorbidity level					
RUB^a^ 3 (low)	20.7 % (258/1244)	14.3 % (1032/7234)	79.5 % (1327/1669)	71.3 % (6187/8683)	44.3 % (2042/4608)
RUB 4	23.8 % (114/479)	12.5 % (253/2028)	89.6 % (583/651)	81.2 %* (2439/3005)	46.3 % (688/1486)
RUB 5 (high)	19.6 % (46/235)	14.5 % (141/971)	93.5 % (272/291)	86.5 %* (1162/1344)	46.9 % (323/689)
Income level					
1 (low)	13.4 % (128/953)	12.7 % (454/3577)	76.1 % (669/879)	68.8 % (3367/4895)	38.4 % (998/2602)
2	18.7 %* (159/851)	13.0 % (451/3481)	72.9 % (679/932)	67.4 % (3433/5092)	39.4 % (1054/2673)
3	24.4 %* (118/484)	13.1 % (427/3263)	76.3 % (595/780)	70.3 % (2893/4118)	42.6 % (881/2067)
4 (high)	19.8 %* (62/313)	12.8 % (342/2681)	79.3 % (452/570)	70.9 % (1766/2492)	42.1 % (672/1597)

^a^Resource utilization band

*Significant difference in chi^2^-test, *p* < 0.05. Multimorbidity level 3 was compared with level 4 and 5 one by one. Income level 1 was compared with level 2, 3 and 4 one by one

Patients 80 years and above had the lowest odds ratios of having indication for treatment (OR 0.66 (CI 95 % 0.54-0.82)), and patients in the second highest RUB level had the highest odds ratios of having indication for the antipsychotic drugs (OR 3.93 (CI 95 % 2.29-6.75)) (Table 5).

**Table 5:** Odds ratio of having indication for treatment (full model)

	Antipsychotics	Proton pump inhibitors	Digoxin	Loop diuretics	Serotonin reuptake inhibitors
	OR (CI 95 %)	OR (CI 95 %)	OR (CI 95 %)	OR (CI 95 %)	OR (CI 95 %)
Gender					
Male	1	1	1	1	1
Female	0.78 (0.62-0.98)	0.99 (0.89-1.11)	1.19 (0.98-1.45)	1.04 (0.97-1.12)	0.83 (0.75-0.91)
Age					
65-79	1	1	1	1	1
80+	0.66 (0.54-0.82)	0.82 (0.74-0.92)	0.85 (0.70-1.03)	0.97 (0.90-1.04)	0.61 (0.56-0.67)
Multi-morbidity level					
RUB^a^ 3 (low)	3.18 (1.90-5.32)	2.86 (2.07-3.96)	7.16 (5.31-9.67)	4.09 (3.60-4.65)	3.00 (2.47-3.65)
RUB 4	3.93 (2.29-6.75)	2.49 (1.76-3.52)	15.7 (10.8-22.8)	7.11 (6.11-8.26)	3.42 (2.76-4.23)
RUB 5 (high)	3.07 (1.69-5.56)	2.99 (2.07-4.31)	26.4 (15.4-45.4)	10.5 (8.63-12.8)	3.58 (2.81-4.55)
Income level					
1 (low)	1	1	1	1	1
2	1.56 (1.20-2.02)	1.04 (0.90-1.19)	0.84 (0.66-1.06)	0.93 (0.85-1.02)	1.08 (0.97-1.21)
3	2.10 (1.57-2.80)	1.02 (0.88-1.18)	0.87 (0.67-1.12)	1.00 (0.91-1.11)	1.17 (1.04-1.33)
4 (high)	1.51 (1.06-2.13)	0.99 (0.84-1.16)	1.00 (0.74-1.34)	1.00 (0.89-1.12)	1.12 (0.98-1.29)

^a^Resource utilization band

### Proton pump inhibitors

Of the patients who used proton pump inhibitors (PPIs), 12.9 % (1 674 of 13 002) had indication for the dispensed PPIs. There was an age difference, where the oldest patients to a lesser extent had indication for the dispensed PPIs. There were no differences in the rest of the subgroups (Table 4).

Patients 80 years and above had the lowest odds ratios of having indication for treatment (OR 0.82 (CI 95 % 0.74-0.92)), and patients in the highest RUB level had the highest odds ratios of having indication for the dispensed PPIs (OR 2.99 (CI 95 % 2.07-4.31)) (Table 5).

### Digoxin

Of the patients who used digoxin 75.8 % (2 395 of 3 161) had indication for the dispensed digoxin. There were no differences in the subgroups (Table 4).

Patients in the second to lowest level of income had the lowest odds ratios of having indication for treatment (OR 0.84 (CI 95 % 0.66-1.06)), and patients in the highest RUB level had the highest odds ratios of having indication for the dispensed digoxin (OR 26.4 (CI 95 % 15.4-45.4)) (Table 5).

### Loop diuretics

Of the patients who used loop diuretics 69.0 % (11 459 of 16 597) had indication for the dispensed loop diuretics. Higher levels of multimorbidity among the patients were to a higher extent associated with indication for treatment (Table 4).

Patients in the second lowest level of income had the lowest odds ratios of having indication for treatment (OR 0.93 (CI 95 % 0.85-1.02)), and patients in the highest RUB level had the highest odds ratios of having indication for the loop diuretics (OR 10.5 (CI 95 % 8.63-12.8)) (Table 5).

### Serotonin reuptake inhibitors

Of the patients who used serotonin reuptake inhibitors (SSRIs), 40.3 % (3 605 of 8 939) had indication for the dispensed SSRIs. The oldest patients had to a lesser extent indication for the SSRIs. There were no differences in the rest of the subgroups (Table 4).

Patients 80 years and above had the lowest odds ratios of having indication for treatment (OR 0.61 (CI 95 % 0.56-0.67)), and patients in the highest RUB level had the highest odds ratios of having indication for the dispensed SSRIs (OR 3.58 (CI 95 % 2.81-4.55)) (Table 5).

## Discussion

On average, less than half of the dispensed drugs had indication for treatment, and there was considerable variation between the different ATC groups of prescription drugs. Proton pump inhibitors were associated with the lowest level of indication for treatment, and digoxin was associated with the highest level of indication for treatment. The oldest patients had the lowest odds ratios of having indication for treatment, and patients with the highest morbidity had the highest odds ratios of having indication for treatment.

It is alarming that so few of the dispensed drugs had correct indications. This is, however, consistent with other studies, showing that between 44-57 % of the patients had at least one unnecessary drug, with lack of indication as one of the most common reasons [27, 28].

One explanation for the very low level of indication for the prescription drugs in this study could be that physicians fail to register diagnoses on their patients. In 2005 and 2006 Östergötland County did not use ACG Case-Mix for reimbursement. Thus, there was no financial, but only a medical incentive to register correct diagnoses, which may have led to deficient registration of diagnoses. Even though the registration may be satisfactory, it is probably not totally complete, and therefore it is likely that the results in this study to some extent may be explained by substandard registration of diagnoses.

Poor planning, evaluation and ending of drug treatment may, furthermore, explain the results. This may in turn be an effect of the organisation of the Swedish Healthcare System. In case of healthcare problems, patients in Sweden are supposed to get in contact with their general practitioner (GP). However, patients may seek secondary care without referral. Whenever this is the case, the liability regarding the prescription of drugs accompanies the patients, regardless of whether or not the patient was referred. Elderly patients often have many caregivers, for example primary healthcare centres, acute care hospitals and rehabilitation departments [29]. Drug therapy is thus initiated at different levels of health care. When non-GP caregivers initiate drug therapy, it is not uncommon to assign the responsibility for evaluation of the prescription drug effect to the patient’s GP. In such cases of lacking continuity in the care of the patients it is important that information about the indication and expected effects, as well as whether the drug therapy is temporary or continuous, is passed on to the GPs. Unfortunately the communication concerning the patients, when transferred between different caregivers, has been shown to be inadequate [30]. Nevertheless, it is hard for any physician to evaluate the effect of a prescription drug not initiated by them, and it is not strange to assume that GPs feel uncertain about evaluating prescription drugs initiated by specialists and vice versa, situations that may lead to unnecessary drug therapy. Increased stress in the health care system and shorter patients consultations [31] may contribute to poorer evaluation and ending of drug therapy [32]. In particular, when it comes to ending drug therapy the physician needs to allocate time to inform the patient about possible scenarios and any follow-up, and this should, furthermore, be well planned to ensure that the patient could turn to the physician in case of any issues or questions.

It is worrisome that the oldest patients in the study population had the lowest odds ratios of having indication for their prescription drugs, since this may lead to unnecessary treatment, and, if the patient use many prescription drugs, preventable polypharmacy. Polypharmacy may be defined as using at least five drugs on a daily basis [33]. It increases the risk of adverse drug events (ADEs) [34], which includes side effects of prescription drugs, lack of effect and interactions between different prescription drugs, and, furthermore, polypharmacy increases the risk of inappropriate prescribing [35]. These effects altogether increase the medical care need, which at worst could lead to hospital admissions [36, 37]. Pharmacological treatment of elderly patients needs to be handled with special care. Elderly patients, who often have a high level of multimorbidity, should probably not be treated according to all clinical protocols and guidelines associated with their diagnoses because of the great risk of developing polypharmacy. Instead physicians need to consider the context the elderly patients live in, together with the overall clinical situation, and plan the pharmacological treatment according to this, an approach that would likely lead to more satisfactory pharmacological treatment among the elderly [38, 39].

Proton pump inhibitors (PPIs) were associated with the lowest level of indication. Only 12.9 % of the prescribed PPIs had an indication. PPIs have in former studies been highly associated with inappropriate prescribing among the elderly with lack of indication as the major problem [40, 41]. In another study 85.8 % of the prescribed PPIs lacked indication, which is fully consistent with our results [42]. PPIs are associated with lack of indication to such a high extent that one may suppose that there is a widespread off-label use of this prescription drug. One example of this could be prescription of PPIs together with NSAIDs, acetylsalicylic acid and steroids, to reduce the risk of developing ulcer in the stomach or duodenum.

Serotonin reuptake inhibitors (SSRIs) and antipsychotic drugs have the lowest odds ratios of having indication for treatment among the oldest patients. Both SSRIs and antipsychotics have in previous studies been associated with high levels of inappropriate prescribing [43, 44]. One explanation for this may be a pronounced off-label prescribing, which is supported by a French qualitative study, where GPs were found to prescribe SSRIs for a wide range of diagnoses other than depression and anxiety disorders [45]. A clinical observation that may further partly explain the low level of indication for SSRIs is that patients using SSRIs and not having any side effects are perceived to be quite reluctant to discontinue their prescription drug, which may contribute to a low level of indication for treatment.

### Limitations

This study is highly dependent on a reliable registration of diagnoses. As mentioned before, substandard registration of diagnoses would lead to lower levels of indication for treatment, with the risk of overestimating the lack of indication for treatment. To increase the reliability we included information about the registered diagnoses for two years. There is furthermore a risk that patients who had a consultation in late December 2004 did not collect their prescription drugs at the pharmacy until January 2005. In this case the collected prescription drug(s) would improperly be categorised as without correct indication, since the diagnoses are registered in 2004.

We have used a well-characterized data set, which have been used in many other studies. Changing prescribing patterns has been proven to be quite hard, even in cases when efforts have been made to accomplish a change [46, 47]. There has been no such effort in Östergötland County, from where the data are collected, and thus there is no reason to believe that the results differ from today.

The information on prescription drugs from the Swedish Prescribed Drug Register contains information on prescription drugs dispensed from the pharmacies and should not be confused with drugs actually prescribed by the physicians. Because of the risk of non-adherence, i.e. patients do not collect all of their prescription drugs from the pharmacies, there is a risk that the results on lack of indication in this study are underestimated.

In this study we were not able to examine the off-label use of the studied prescription drugs. High levels of off-label use, which as stated above has been reported for both PPIs and SSRIs, may have led to lower levels of indication for treatment, but at the same time not necessarily to unjustified pharmacological treatment.

It would have been interesting to examine inappropriate prescription following the Beers Criteria, even though it is beyond the scope of this study, but we had no access only to data from the CDWÖ register, containing no information on Beers Criteria. Furthermore we had no access to the longitudinal data required to relate health outcomes or adverse drug reactions to the outcome.

## Conclusion

On average, less than half of the studied prescription drugs had indication for treatment. The quality was highest in relation to multimorbidity and lowest in relation to age, with the lowest rates and odd ratios of having indication for treatment among the oldest patients. Since lack of quality of prescription drug use is highly associated with inconvenience among the elderly, as well as high costs to society, it is important that future research and allocation of resources focus on the quality of elderly patients’ drug therapy. In the end, this could for example lead to complete and up-to-date prescription drug lists, longer doctors visits for elderly patients and more time for evaluation of prescription drug use among elderly patients.

## Abbreviations

ACG: Adjusted Clinical Groups
ATC: Anatomical therapeutic chemical
CDWÖ: Care Data Warehouse Östergötland
CI: Confidence interval
GP: General practitioner
NSAID: Non steroidal anti-inflammatory drug
OR: Odds ratio
PPI: Proton pump inhibitor
RUB: Resource utilization band
SSRI: Serotonin reuptake inhibitor
